# Stem cells in tissues, organoids, and cancers

**DOI:** 10.1007/s00018-019-03199-x

**Published:** 2019-07-17

**Authors:** Xusheng Wang

**Affiliations:** School of Pharmaceutical Sciences (Shenzhen), Sun Yat-sen University, Guangzhou, 510275 China

**Keywords:** Stem cell, Stem cell niche, Organoid, Cancer stem cell

## Abstract

Stem cells give rise to all cells and build the tissue structures in our body, and heterogeneity and plasticity are the hallmarks of stem cells. Epigenetic modification, which is associated with niche signals, determines stem cell differentiation and somatic cell reprogramming. Stem cells play a critical role in the development of tumors and are capable of generating 3D organoids. Understanding the properties of stem cells will improve our capacity to maintain tissue homeostasis. Dissecting epigenetic regulation could be helpful for achieving efficient cell reprograming and for developing new drugs for cancer treatment. Stem cell-derived organoids open up new avenues for modeling human diseases and for regenerative medicine. Nevertheless, in addition to the achievements in stem cell research, many challenges still need to be overcome for stem cells to have versatile application in clinics.

## Introduction

The term stem cell (SC) appears in the scientific literature as early as 1868 in a publication written by Ernst Haeckel. However, it was only in the 1960s that definitive evidence of the existence of SCs in the hematopoietic system was provided, in which Till and McCulloch described the existence of clonogenic bone marrow (BM) precursors that generate macroscopic spleen colonies after being injected into irradiated recipient mice. Based on this eminent work, the two gold standard features of SCs were proposed: an SC is capable of long-term self-renewal and multilineage differentiation [1]. SCs residing in our body are responsible for the maintenance of tissue homeostasis; however, identifying definite markers for SCs in vivo remains the biggest challenge in the field of SC research. Recent advances in lineage tracing have enabled the genetic labeling of a single cell or a set of cells in a normal physiological context and is a powerful method for assaying the contribution of SCs to tissue in homeostasis or disease [2]. Moreover, the establishment of embryonic stem cells and induced pluripotent stem cells highlight the potential of stem cells’ application in regenerative medicine.

## Diversity and heterogeneity of stem cells in tissue

Several SCs and their niches in mammalian tissues have been identified, including hematopoietic SCs (HSCs) in the BM [3], germline SCs in the seminiferous tubules basal layer [4], epithelial SCs in the basal layer of the epidermis and the bulge of hair follicles [5], neural SCs (NSCs) in the lateral ventricle subventricular zone (SVZ) of the central nervous system [6], and muscle SCs among satellite cells under the basal lamina of myofibres [7].

### Hematopoietic stem cells

**Fig. 1:**
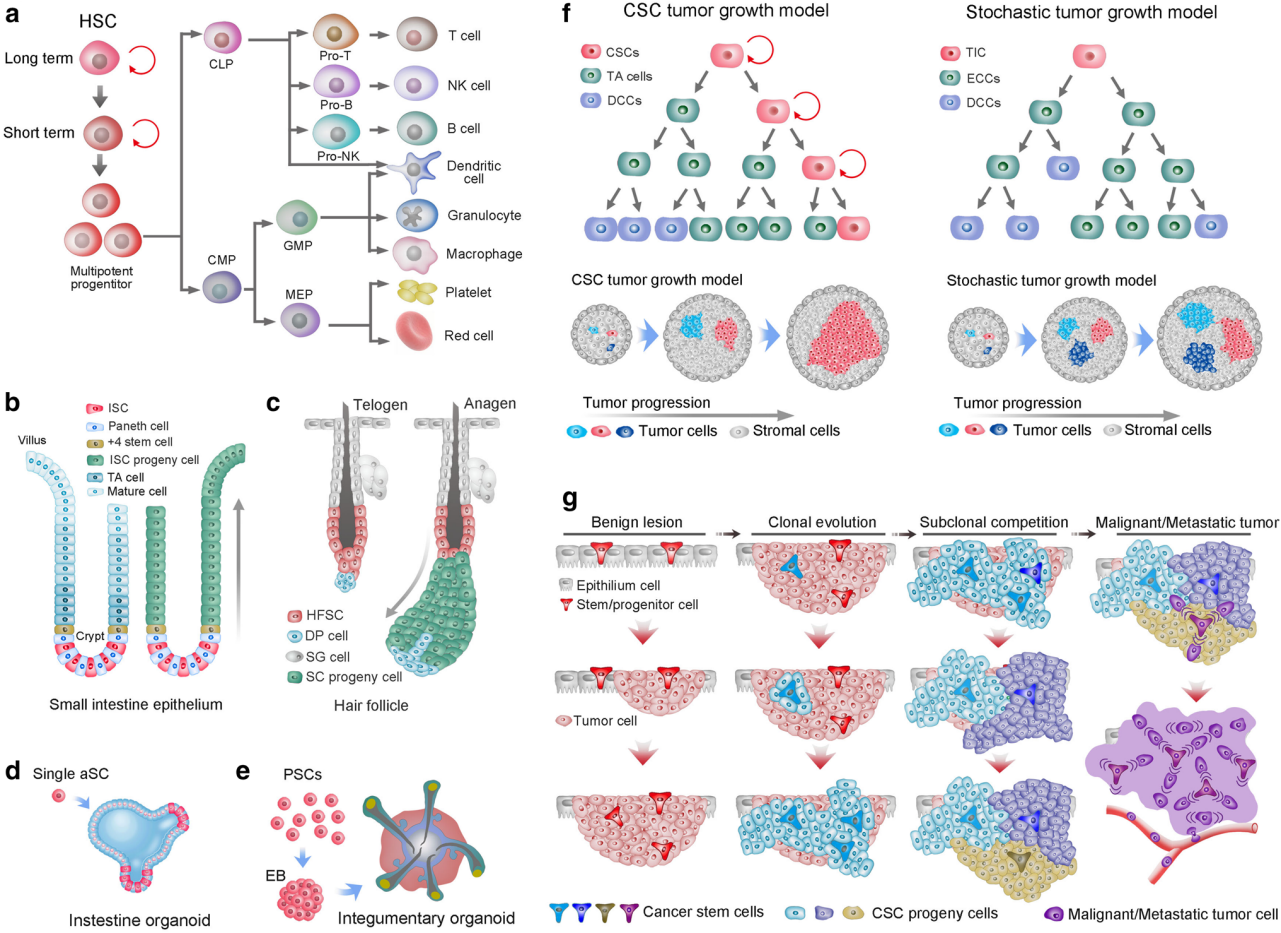
Stem cells in tissues, organoids, and cancers. **a** Differentiation of hematopoietic stem cells (HSCs). HSCs are composed of long-term and short-term self-renewing stem cells and multipotent progenitors. The multipotent progenitors give rise to common lymphoid progenitors (CLPs) and common myeloid progenitors (CMPs). Subsequently, CMPs and CLPs develop into myeloid and lymphoid lineages of blood cells, respectively. Both CMPs and CLPs can generate all dendritic cells in mice. GMPs, Granulocyte macrophage precursors. **b** Intestinal stem cells (ISCs) at the base of the crypt generate rapidly proliferating TA cells in the lower half of the crypt. TA cells subsequently differentiate into the mature lineages of the surface epithelium (left). Lineage tracing showed that ISCs could repopulate the epithelium in 5–7 days (right). **c** The hair follicle stem cells (HFSCs) reside in the bulge region and maintain quiescence during the telogen phase (left). HFSCs generate all cycling portions of hair follicles in the anagen phase (right). **d** Single Lgr5 stem cell from small-intestinal crypts build crypt villus organoids in 3D culture. **e** A homogeneous population of mouse pluripotent stem cells generates skin organoids in vitro, which stratify with epidermal and dermal layers, and generates de novo hair follicles in a process that recapitulates embryonic hair folliculogenesis. **f** Two models of tumor growth. In the hierarchical model of tumor growth, only CSCs exhibit self-renewal capacity, whereas TA cells confer limited proliferative potential and subsequently differentiate into nonproliferative cancer cells (left upper). In the clonal assay, CSCs present dominant clonal expansion, whereas TA cells exhibit limited clonal expansion capacity (left lower). In the stochastic model of tumor growth, all cancer cells are equipotent and undergo either self-renewal or differentiation into nonproliferative cancer cells stochastically (right upper). In the clonal assay, all equipotent cancer cells showed similar clonal expansion capacity (right lower). **g** The hypothesis of clonal evolution in tumor progression. First, oncogenic stimulation insults a stem cell (alternatively, a progenitor or even a differentiated cell) of healthy epithelium, resulting in the generation of benign lesions with genetic homogeneity (benign lesion). Further evolution of the cells in the benign lesion generates a more invasive and malignant clone in the primary tumor (clonal evolution). Subsequently, subclone competition within the malignant subclones leads to further transformation, and genetically heterogeneous subclones coexist within the tumor (subclonal competition). Then, a final mutational insult leads to the tumor being thoroughly turned over by the malignant and metastatic cells that all behave as cancer stem cells

HSCs are probably the best-characterized SC population and have been shown to give rise to both myeloid and lymphoid lineages of blood cells (Fig. 1a). Mice hematopoietic progenitors can be found at 8–8.5 days postconception in the yolk sac and the embryo proper, after which they shift to the spleen and then begin to shift to the BM before birth [8]. By birth in humans, HSCs in BM support the vast majority of hematopoiesis. In contrast, the kidney is predominantly responsible for hematopoiesis in adult fish [9]. Not all HSCs are alike; they have various physical characteristics, including cell cycle status, cell surface marker phenotype, response to different extrinsic signals and different lineage outputs following transplantation. HSC subtypes can be classified into long-, intermediate- and short-term repopulating HSCs based on the reconstitution kinetics following clonal HSC transplantation [10]. Despite many exhaustive studies, researchers have yet to find a single molecular marker that is expressed exclusively by HSCs. Furthermore, HSCs do not express any lineage-specific antigen; thus, they are referred to as lineage-negative cells [11]; while HSCs can be distinguished from mature blood cells by the presence of certain other cell-surface antigens, such as c-kit and Sca-1 (for murine cells), CD133 and CD34 (for human cells) [12]. CD34 was the first differentiation marker to be recognized on primitive human hematopoietic cells and is still the most commonly used marker to obtain enriched populations of human HSCs and progenitors [13]. Antigens such as CD90 and CD117 are also expressed by HSC. In line with their immaturity, HSCs do not express CD38, CD45RA, CD71, HLA-DR [14]. The heterogeneity within HSCs makes HSCs the most robust cell fraction at the foundation of the hematopoietic system; it is currently of great interest and raises questions as to why HSC heterogeneity exists, how HSCs are developed and whether HSC heterogeneity is relevant to leukemogenesis or treatment options.

### Gut stem cells

The most frequently self-renewing tissue in mammals is probably the intestinal epithelium, which is replaced every 4–5 days. This homeostasis is maintained by the intestinal SCs (ISCs) that are retained in the bottom of crypt-like invaginations and divide every 24 h [15]. Lgr5 was identified as a specific marker of ISCs, which give rise to all functional cells of the villi [16]. An alternative DNA label-retaining crypt SC is located at the + 4 position, just above the Lgr5+ SC niche and features Bmi1 expression (Fig. 1b). Bmi1+ cells expand following depletion of Lgr5+ cells to replace the loss of the actively cycling SC pool [17]. Secretory precursor cells reside just above the + 4 position SC zone and express Dll1. During homeostasis, Dll1+ cells generate small, short-lived clones that contain Paneth, goblet, enteroendocrine and tuft cells. However, the same Dll1+ cells can regenerate entire crypt–villus units once the SC pool is ablated by irradiation [18]. Paneth cells are secretory cells that serve as niche cells providing Lgr5+ SCs with Wnt, Notch and EGF signals [19]. However, this quiescent SC niche cell could also be endowed with a SC state and generate clones comprising the main cell types of intestinal epithelial cells. These collective studies induce debate over the precise identity and function of ISCs, and it is becoming apparent that there is probably no definitive answer. Together, the intestine seems capable of activating several highly plastic reserve SC populations in the lower regions of the crypt to maintain epithelial homeostasis and affect tissue regeneration following injury, rather than relying on a single and rigid SC compartment.

### Hair follicle stem cells

The hair follicles provide excellent models for the study of SC biology. Hair follicles consist of a permanent bulge region and a cycling portion that cycles through anagen (growth phase), catagen (retraction phase), and telogen (resting phase). Hair follicle SCs (HFSCs) were first described as label-retaining cells (LRCs) in the bulge region. Elaine Fuchs and colleagues found that the majority of LRCs expressed the SC marker CD34 (Fig. 1c). The sorted and cultured CD34+ bulge cells were shown to regenerate the entire hair follicle after transplantation [20, 21]. However, the bulge SCs were found to not directly generate TA cells but rather give rise to the Lgr5+ cell population located at the hair germ, and the hair germ cells in turn generated TA cells that further differentiated into the hair shaft in the hair matrix [22, 23]. Further study determined that the Lgr5+ cells in hair germ were cycling yet long-lived and functional HFSCs, which are capable of generating new hair follicles and give rise to all cell lineages of the cycling portion of hair follicles. After being activated during hair follicle anagen, Lgr5+ progeny repopulate CD34+ bulge SC compartments [24]. Thus, hair follicles seem to maintain a heterogeneous SC pool that contains both quiescent and active SC populations in separate yet adjacent locations. The resting phase of the hair follicle is synchronous in mice and can last months. During this time, HFSCs in bulge hair germ are quiescent.

### Stem cells in organoids

Organoids are SCs that form 3D structures which consist of organ-specific cell types and self-organize through spatially restricted lineage commitment. Both pluripotent SCs (PSCs) and restricted adult SCs (aSCs) can initiate organoids. Organoids can start, but are not essential, from a single aSCs, while cell aggregation is typical starting material for PSC-derived organoids. As a receptor for the secreted R-spondins and Wnt target gene, Lgr5 marks active aSC in many epithelia, and Lgr5+ aSCs exhibit a high capacity to form organoids. Lgr5+ crypt SCs were first established to grow epithelial organoids in culture as ‘‘mini-guts’’ (Fig. 1d). Organoids of the stomach, liver, pancreas, mammary gland and taste buds from single Lgr5+ SC of the respective tissue were subsequently established [25]. Organoids of lung, fallopian tube, salivary gland and prostate can also grow from single cell, but not Lgr5+ cells [26–29]. PSCs, including embryonic SCs (ESCs) and their synthetic induced PSC (iPSC) counterparts, can generate more complicated organoids, such as brain organoids. Additionally, organoids of the lung, kidney, inner ear and skin complex can also be generated from PSCs [30, 31]. Unlike single aSC that build organoids, PSC-derived organoids develop with a spheroid or embryonic body intermediate stage. Activin treatment of PSCs generates definitive endoderm, subsequent inductive signals instruct cells to various endodermal organ identities, including organoids of the stomach, lung, thyroid and small intestine [25]. The iPSC-derived skin organoids spontaneously produce hair follicles in a process that mimics normal embryonic hair folliculogenesis, and the hair follicle developed in skin organoids contains HFSC niches containing cells with HFSC markers [32]. Equally developed skin organs in vivo even produce hair follicles that showed proper hair eruption and hair cycles [33] (Fig. 1e). This renders hair follicles the first authentic and functional mini-organ in PSC-developed organoids.

### Cancer stem cells

The cancer SC (CSC) concept was born from the observation of explicit histological heterogeneity in tumors and the observation that a new tumor could initiate from a single mouse tumor cell [34]. In the early nineties, Dick and colleagues observed that most subtypes of acute myeloid leukemia (AML) could be engrafted reliably in immunodeficient mice and that leukemic engraftment could only be initiated from CD34^+^CD38^−^ fractions. Thus, a CSC was identified in AML. Moreover, the xenograft assay measured the frequency of the initiating cell to be one per million (~ 1/10^6^) tumor cells [35]. However, the interpretation of these xenotransplantation studies is challenged by studies that infused titrated numbers of mouse tumor cells into nonirradiated histocompatible recipient mice. These studies of mouse lymphomas and leukemias indicate that malignancies can be maintained by a relatively large proportion (> 10%) of tumor cells. Accordingly, some human cancers may be found that do not adhere to the CSC hypothesis. Thus, the CSC concept, albeit imperfect, encompasses the notion that this cell type sustains the growth of many cancers and possesses SC properties, such as the capacity for self-renewal and giving rise to “differentiated” progeny [36, 37]. Solid tumors, such as breast cancer, brain cancer, prostate cancer, pancreatic cancer, colon cancer, lung cancer, and ovarian cancer, have been shown to contain subpopulations of tumor cells with great ability to propagate tumors in xenotransplantation assays [38]. In addition, studies of distinct mouse models of tumors have validated that tumors contain cells that act as CSCs and substantially fuel tumor growth, irrespective of the oncogene utilized and the tissue origin of these tumors [39–41]. Consistently, a predominant clone could be detected in every sequenced tumor tissue, supporting a selective advantage conferred by independent mutation endowing a clone to outstrip other clones [42, 43] (Fig. 1f). However, the CSC concept is still contentious, namely, there is no consensus on whether CSCs are rare or high-frequency cells or whether they have fixed, hierarchical or diverse phenotypes [44–46]. Modern cancer biology and genome sequencing have identified cancer as a complex, Darwinian and adaptive tissue ecosystem [47] (Fig. 1g). Thus, given the evolutionary progression, clonal expansion and genetic heterogeneity in cancer, CSCs are unlikely to be fixed entities. Nevertheless, the relationship and dynamics among CSC-derived predominant clones and other minor clones within a tumor remain poorly described.

## Niches of stem cells

The SC niche is defined as a microenvironment that anchors SCs to maintain their stemness. Precise control over SC differentiation and tissue architecture is essential for development, organogenesis and tissue homeostasis. A niche is defined by anatomy and function—a local tissue microenvironment that directly maintains and regulates a particular type of SC or progenitor.

### Niche of HSCs

**Fig. 2:**
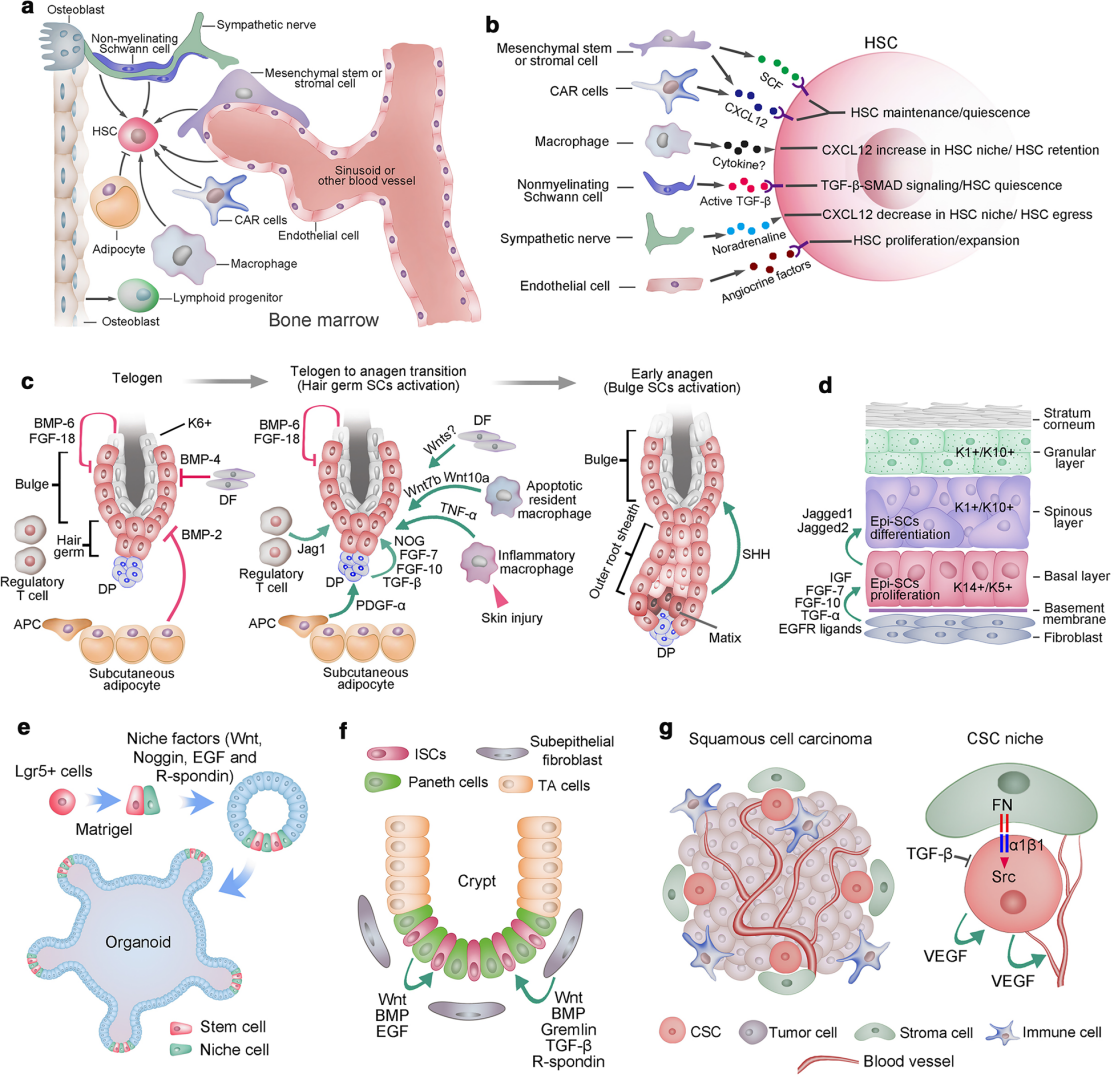
Stem cell niche. **a.** Various cell types in bone marrow play roles in regulating HSC maintenance, including mesenchymal stem/stromal cells, endothelial cells, CAR cells, macrophages, sympathetic neurons and nonmyelinating Schwann cells. However, adipocytes exhibit a negative effect on HSC maintenance. **b.** HSC niche cells contribute to HSC maintenance via the release of different factors. **c.** The quiescence of hair follicle SCs at the bulge and hair germ was maintained by a set of factors, including BMP6 and FGF-18 from K6+ bulge cells, BMP4 produced by dermal fibroblasts (DFs), and BMP2 expressed by subcutaneous adipocytes. At the onset of anagen, the activation factors prevailed, including noggin (NOG), FGF-7, FGF-10 and TGF-β2 produced by dermal papillae (DP) and PDGF-α derived from adipocyte precursor cells (APCs). Wnt7b and Wnt10a from apoptotic resident macrophages and Jag1 from regulatory T cells also contribute to the activation of hair follicle SCs. After skin injury, TNF-a from inflammatory macrophages could induce the activation of hair follicle SCs. At the beginning of a new hair cycle, SCs in the bulge remain quiescent until SHH is expressed by the TAC matrix. **d.** The epidermis is a stratified structure that is composed of the basal cell layer and the underneath basement membrane, spinous layer, granular layer and stratum corneum layer. Self-renewing and proliferating epidermal stem cells are located within the basal layer. Secreted factors, such as dermal fibroblasts, promote the self-renewal of cells in the basal layer. These factors include IGF, FGF-7, FGF-10, EGF ligands and TGF-α. Epidermal stem cells generate columnar units that undergo terminal differentiation via jagged activated Notch signaling. **e.** The essential components for intestine Lgr5+ stem cells to generate self-renewing epithelial organoids in vitro, including laminin-rich Matrigel, a cocktail of niche factors including Wnt, Noggin, R-spondin 1 and EGF that recapitulate the ISC niche in vivo. **f.** Paneth cells and subepithelial fibroblasts at the crypt bottom constitute the niche for intestinal stem cells. **g.** The CSC niche of squamous cell carcinoma. CSCs of squamous cell carcinoma are frequently found at the tumor-stroma interface (left). Extracellular matrix ligands, such as fibronectin (FN), could activate αβ1 integrins, resulting in hyperactivated focal adhesion kinase (FAK) and its associated tyrosine kinase Src, leading to the proliferation of CSCs. However, TGF-β signaling maintains the quiescence of CSCs. Moreover, VEGF secreted by CSCs could enhance CSC proliferation and promote the formation of new blood vessels

Notable efforts have been made to uncover the regulatory mechanisms that maintain HSC niches. HSCs are found mainly around the sinusoids throughout the BM, where mesenchymal stromal or stem cells (MSCs) and endothelial cells contribute to HSC maintenance [48, 49] (Fig. 2a). Although perivascular MSCs are likely to be heterogeneous, they generate osteoblastic cells and synthesize factors (such as Scf and Cxcl12) that promote HSC maintenance [50]. Ablation of MSCs results in BM hypocellularity, anemia and attenuation of osteogenic cells [51, 52]. CXCL12-abundant ‘reticular’ (CAR) cells were found to colocalize with HSCs around the sinusoids [53]. CAR cells play a crucial role in promoting HSC maintenance, as ablation of CAR cells depletes HSCs and severely decreases the osteogenic and adipogenic capacities of BM cells [54]. CD146+ skeletal SCs in the human BM also contribute to the HSC niche by synthesizing high levels of factors such as SCF and CXCL12 [55]. Although CXCL12 and SCF have been established as regulators of HSC maintenance, other HSC niche regulatory factors have also been identified, such as pleiotrophin, angiopoietin-1, and Notch and Wnt in TGF-β signaling pathways [56] (Fig. 2a). BM endothelial cells line the surface of blood vessels and promote HSC maintenance during homeostasis and regeneration after injury [57]. Further study found that endothelial cells are capable of promoting hematopoiesis via the expression of essential surface markers such as E-selectin [58] and upregulation of ‘angiocrine’ factors such as FGF2, DLL1, DHH and EGF [59, 57, 60]. Trafficking of HSCs into the bloodstream in the BM is closely associated with the adrenergic signals from the sympathetic nerves, indicating that sympathetic nerves could modulate HSC function [61]. Macrophages have been identified as the key niche-regulating cells given their effect on nestin-expressing niche cells by inducing CXCL12 secretion, which in turn promotes HSC retention [62]. Other cell types that regulate HSC niches include myelinating Schwann cells, adipocytes and osteoclasts [63, 64]. In addition, extracellular matrix and calcium play a role in regulating HSCs (Fig. 2b). Unlike SCs in some other tissues, HSCs cannot be stably expanded in vitro. This greatly attenuates the potential of HSC transplantation in certain clinical contexts. Further elaboration on how the microenvironment participates in HSC regulation in normal and disease physiology will provide new strategies for hematological disorders.

### Niche of hair follicle stem cells

HFSCs maintain a quiescent state that is synchronous with the rest phase of the hair cycle and only activate in the anagen of hair follicles. At the telogen phase, dermal fibroblasts secrete BMP4, subcutaneous fat produces BMP2, and the BMPs contribute mainly to the quiescence state of HFSCs [65]. In addition, the inner bulge layer expresses high levels of BMP6 and FGF-18 (another quiescence factor). Together, these factors maintain the quiescence of HFSCs in both bulge and hair germ [66]. In contrast, the dermal papillae (DP) beneath the hair germ is an indispensable niche component that induces HFSC activation [67]. The DP-derived HFSC-activating factors include TGF-β2, FGF-7, FGF-10, and noggin [68, 69]. In addition, adipocyte precursor cells underlying the dermal layer produce platelet-derived growth factor-α (PDGF-α), which could also contribute to the activation of SCs in hair germ [70]. WNT signaling counteracts BMP signaling to determine the activation and quiescence state of hair follicles. Nuclear β-catenin accumulates in the activated hair germ SCs, and β-catenin-knockout hair follicles arrest in telogen [71]. However, the exact cues of Wnt signaling remain unknown, and hair germ and dermal fibroblasts are potential sources of Wnt ligand(s) [22, 72]. Unlike the SCs in hair germ, SCs in the bulge seem to be activated upon alternative signals. There are data indicating that sonic hedgehog (SHH) secreted by the newly formed transient amplifying cell (TAC) matrix in hair germ affects Bu-SC activation. Moreover, the SHH from the TAC matrix induces the expression of noggin and FGF-7d in DP, and these factors promote the proliferative state of the matrix and lower ORS [68, 69]. Together, DP interacts with the hair germ to initiate anagen, and then the signals from the emerging TAC pool activate the SCs in the bulge. Additionally, skin resident macrophages and regulatory T cells also play a role in modulating HFSC activity [73, 74]. The sensory neurons adjacent to the upper bulge play a role in influencing the behavior of HFSCs [75, 76]. Moreover, during mouse skin injury, TNF-α derived from inflammatory macrophages could activate SCs in hair germ and induce hair follicle regeneration [77, 78] (Fig. 2c). After the activation of HFSCs and a predictable period of time anagen phase, follicle growth stops, and catagen begins. The catagen phase is a highly controlled process of coordinated cell differentiation and apoptosis. Upon catagen initiation, the molecules that could serve as anagen-supporting signals, include insulin-like growth factor I receptor (IGF-IR) and keratinocyte growth factor (KGF), are downregulated, while BDNF and TGF-β1 are upregulated [79]. After the catagen phase, the progeny of HFSCs in hair germ repopulate bulge SC compartments. In the telogen phase of the hair follicle, HFSCs are kept quiescent and can last months. Fgf18 has been found to prevent anagen entry and is essential to maintain quiescence of HFSCs [20].Why does the cycle end and when it does? Is this due to the HFSCs being instructed to be quiescent and stop to fuel the transit amplifying cell population? Or increased expression of catagen inducer, including the dickkopf-1 (DKK1), in DP cells? Or, is it due to changes in activities of perifollicular mast cells, regulatory T cell and macrophages [73, 80, 81]? As components of HFSC niche continue to emerge, the interaction between the HFSCs and their niches should yield new insights into how SC heterogeneity is organized in hair follicles and how SCs are regulated to determine which lineage to embark upon.

### Stem cell niche in the interfollicular epidermis

Mammalian skin is covered with a stratified epidermis, and the basal epidermal layer of interfollicular epidermis (IFE) contains epidermal SCs that express keratin5 and keratin14 (K5/K14). These epidermal SCs undergo self-renewal and differentiate into nonproliferative, K1/K10 and involucrin-positive outer epidermal layers [82]. Epidermal SCs proliferate at the basal layer and subsequently move upward and differentiate. Dermal fibroblasts are a rich source for mitogens that stimulate epidermal SC proliferation, including insulin-like growth factors (IGFs), FGF-7, and FGF-10, and especially EGF, which is a crucial signaling pathway for epidermal growth [83]. Consistently, activation of TGF-α or deletion of Mig6, which are the positive and negative regulators of EGFR signaling, respectively, results in epidermal hyperproliferation in mice [84, 85]. Epidermal stratification is achieved first by the detachment of basal cells from the basement membrane and then by the asymmetrical cell division of basal cells, which generates a committed daughter and a proliferative basal cell [86]. The differentiation of basal cells to spinous cells depends on Notch signaling, and Notch1/2/3 receptors and Jagged1 are expressed in the mouse suprabasal epidermis, whereas Jagged2 is expressed in the basal layer cells [87] (Fig. 2d). Consistently, elevated Notch signaling in basal cells leads to massive expansion of spinous cells [88]. However, the cues that trigger Notch signaling in the mouse epidermis and their cellular source require further elaboration. It will be interesting to assay the cellular plasticity between the basal SCs and differentiated spinous cells.

### Dissecting the ISC niche with organoid models

SCs can generate organoids in conditions that mimic the SC niche of physiological SC self-renewal or tissue repair. Thus, organoids could be used as a powerful tool to dissect the niche of SCs. Early studies found that in vivo niche signals, such as Wnt/R-spondin that regulates ISCs and crypt homeostasis, are also indispensable for the in vitro maintenance of ISCs in organoid cultures [89–91]. Moreover, by reconstituting aggregates containing SCs and purified Paneth cells, Sato and colleagues found that Wnt secretion by Paneth, combined with the soluble factors EGF, R-spondin, and Noggin, can serve as a minimal niche for maintaining ISCs in vitro [19]. Although Wnt3-producing Paneth cells are required for the maintenance of Lgr5+ ISCs in vitro, they are dispensable in vivo. The mesenchymal cells that surround SCs could provide an alternative source of Wnt in vivo [92, 93]. As initially described for small ISCs, the Wnt pathway has emerged as the major driver of epithelial aSCs [90, 93]. Lgr5, a receptor for Wnt agonist R-spondins and itself encoded by a Wnt target gene, marks active aSCs in many epithelia. Thus, it is not surprising that Wnt activators are key components of most aSC-derived organoid cultures and that Lgr5+ aSCs invariably present in such cultures, such as organoids of the intestine, stomach, liver and taste buds, are derived from Lgr5+ SCs [25]. In contrast, BMP signaling is responsible for epithelial differentiation and negatively modulates the number of ISCs in the intestine [94]. Additionally, mesenchyme-derived BMP is crucial for intestinal differentiation in vivo; thus, BMP antagonism, such as Noggin, is essential in mini-gut culture [19]. Equally, TGF-β also inhibits intestinal organoid proliferation, and small molecules are exploited for TGF-β inhibition in the culture of intestinal organoids. Thus, inhibition of BMP or TGF-β signals is a prerequisite for organoid growth. In addition, EGF signal activation is required for long-term mini-gut organoid culture, and EGFR inhibitor treatment slows human intestinal organoid growth within a week [95]. In addition, a Notch-positive feedback in the intestinal stem cell niche was proved to be essential for stem cell self-renewal, this highlights the importance of dynamical system analysis and agent-based multiscale stochastic modeling mechanisms in studying the spatiotemporal control of the stem cell niche [96]. The development of organoid techniques will improve our understanding of the SC niche by providing efficient tools that could dissect the SC niche at the single-cell level.

### Cancer stem cell niches

Accumulating data suggest that the growth of at least some cancers is driven by CSCs [97]. Tumorigenic CSCs often share similar phenotypic and functional characteristics with normal SCs in the same tissue [98]. Correspondingly, CSCs might be supported by specialized niches for their maintenance, similar to normal SCs. However, recent effort has focused on identifying markers to clearly distinguish CSCs from other cancer cells; thus, a few CSC niches were partially dissected. CSCs in mouse squamous cell carcinoma (SCC) have been purified and characterized [99]. SCC CSCs are located at the tumor-stroma interface and have high expression levels of integrins [100, 101]. SCC CSC proliferating activities are regulated by signals from their niche, where TGF-β signaling interacts with signals from integrin and focal adhesion kinase to modulate the activity of CSCs [101]. In addition, SCC CSCs produce vascular endothelial growth factor (VEGF), which promotes vascularization in the tumor. Moreover, VEGF may also contribute to the CSC niche and maintain tumor growth by acting on CSCs in an autocrine fashion [102]. Therefore, the self-renewal of CSCs is regulated by both autocrine and paracrine niche signals for self-renewal and differentiation, which is similar to the SC counterparts of CSCs in normal tissue. Even though the HSC niche was extensively explored, the extent to which the leukemia SC niche shares physiological characteristics with the normal HSC niche is still largely unknown. Infiltration of human acute lymphogenous leukemia in immunodeficient mice changes the homing sites of healthy HSCs [103]. The extensive proliferation of cancer cells causes a hypoxic microenvironment within tumors, and the hypoxic niche promotes cancer cells to gain CSC properties. Inhibiting hypoxia-inducible factors in hematological malignancies attenuates the tumor-propagating potential of leukemic and lymphoma CSCs [104]. Studies on brain tumor SCs found that these cells tend to be adjacent to blood vessels more often than other tumor cells, and vascular cells contribute to brain tumor SC maintenance in culture and facilitate tumor development in vivo [105]. Furthermore, MSCs can induce breast cancer cells with CSC properties by elevating miR-199a expression in cancer cells [106]. Notably, a very recent study found that carcinoma-associated fibroblasts promote tumor formation and chemoresistance by providing a survival niche for CSCs [107]. These studies highlight the potential of anticancer therapies to be more efficient than other therapies by targeting the CSC niche in addition to targeting cancer cells themselves.

## Plasticity and transformation of stem cells

The study of cellular plasticity initiated at the beginning of the new century and found that cellular identity could cross developmental germ layers and undergo extreme changes [108]. However, these notable claims were largely based on studies performed in vitro. It is evident that mammalian cells can change their cell identity under ‘natural’ conditions in response to intrinsic changes in the cell or physiological stresses. Even though the exact role of adult cell plasticity in vivo still needs to be evaluated on a case-by-case basis, the presence of many examples of cellular plasticity throughout the animal kingdom suggests that it plays a conserved role during tissue homeostasis and repair.

### Nuclear reprograming and nuclear plasticity

**Fig. 3:**
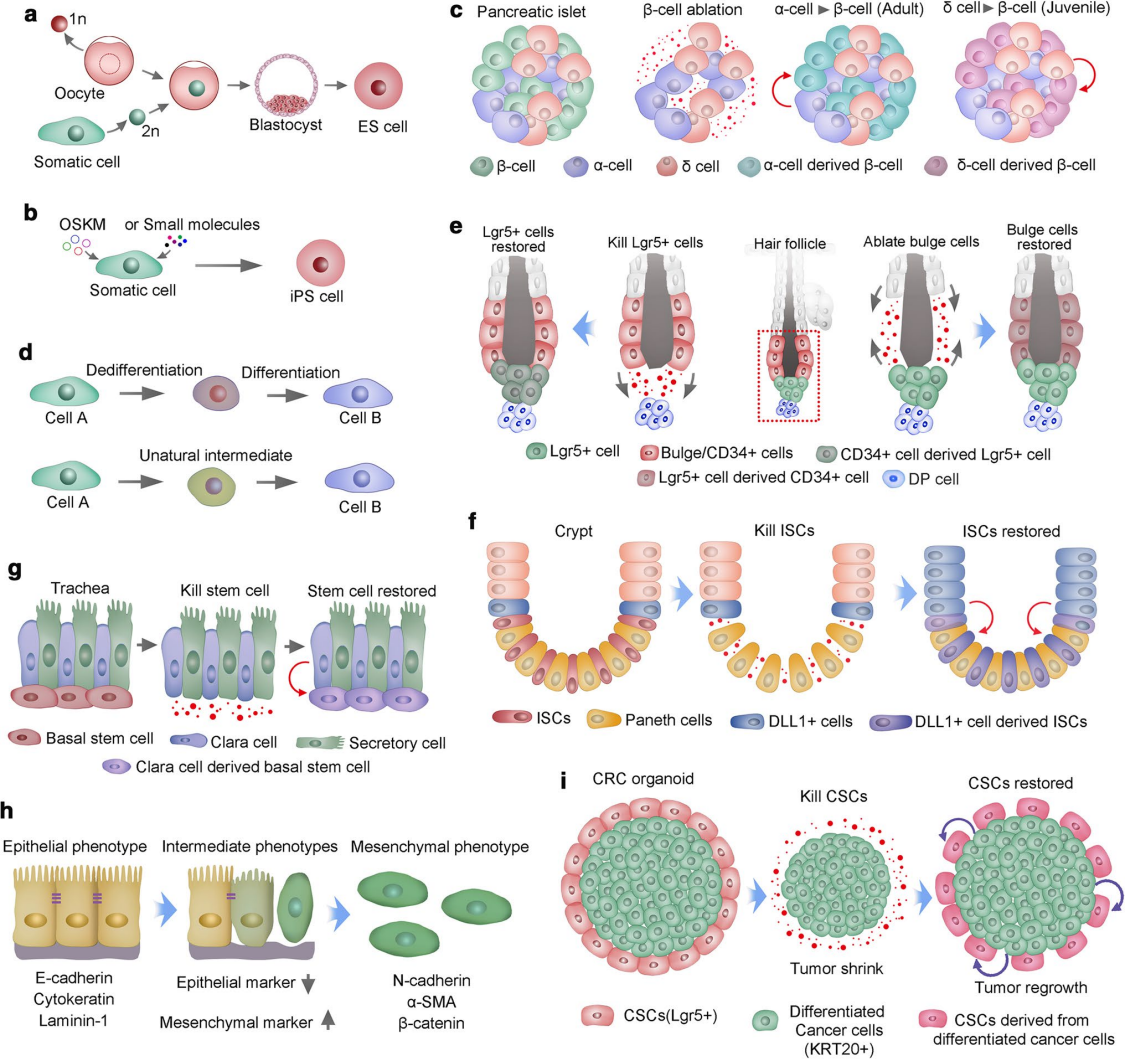
Stem cell plasticity. **a** Somatic cell nuclear transfer (SCNT) for nuclear reprogramming. The nucleus of a somatic cell (diploid, 2n) is transplanted into an enucleated oocyte (haploid, 1n). In the oocyte, the somatic cell nucleus is reprogrammed; thus, the cells derived from it are pluripotent stem cells. **b** Transcription factor transduction (Oct4, Sox2, Klf4 and cMyc, OSKM) or small molecule-induced pluripotent stem (iPS) cells. **c** Two transdifferentiation models. The first model presumes that a cell must first dedifferentiate into a precursor stage before it converts to a lineage. In the second model, cells transdifferentiate to generate new cells directly, in some cases mediated via an unnatural intermediate phase and in which genetic programming of two cell types is simultaneous. **d** Pancreatic islets have β-, α- and δ-cells. In adult mouse islets, α-cells transdifferentiate directly into insulin-producing cells after ablation of β-cells. However, in juvenile islets, δ-cells generate β-cells following ablation of β-cells. **e** During homeostasis, the hair follicle stem cell compartment is maintained by distinct stem cells, and ablated bulge cells (CD34+) can be replenished by cells in both the upper pilosebaceous unit and the hair germ (Lgr5+). Correspondingly, CD34+ bulge stem cells could compensate for the loss of Lgr5+ stem cells in hair germ. **f** Crypt stem cells give rise to all cell lineages in the mammalian intestinal epithelium during homeostasis. Radiation injury ablates ISCs, which stimulate dedifferentiation of DLL1+ cells to generate new ISCs. **g** Basal stem cells in the trachea give rise to differentiated secretory cells and Clara during homeostasis. Ablation of basal stem cells induces the dedifferentiation of Clara cells and generates new basal stem cells; **h** EMT is a transition of polarized epithelial cells into mobile mesenchymal cells. Several commonly used markers of epithelial and mesenchymal cells are listed. **i** Lgr5+ CSCs proliferate and differentiate into KRT20+ CRCs at steady state, depletion of CSCs results in the reduction of tumor size, some KRT20+ cells convert to Lgr5+ CSCs, and tumor regrowth occurs

Somatic cells within a multicellular organism are progressively committed to phenotypically and functionally distinct fates during development. Somatic cells are considered to be stably restricted in differentiated status throughout the lifespan of the organism. In the 1960s, Gurdon transferred the nuclei from highly differentiated tadpole intestinal cells into ultraviolet-light-irradiated oocytes and successfully generated normal adult frogs [109, 110]. Gurdon’s reports provided strong evidence that differentiation might be reversible. Three decades later, Wilmut and colleagues succeeded in a cloning mammal, a sheep named Dolly. A year later, the cloning of mice was accomplished [111]. The process of somatic cell nuclear transfer (SCNT) (Fig. 3a), in addition to being used to clone sheep and mice, was soon used to clone a wide range of other species, such as cattle, dogs, goats, pigs and wolves [112, 113, 36, 114]. However, it was not until 20 years after Dolly was cloned that primates, two cynomolgus monkeys, were cloned by SCNT [115]. Although it is evident that nuclear reprogramming is crucial for the outcome of cloning, various questions remain unanswered regarding the low efficiency of SCNT. Improving the efficiency of SCNT would greatly promote the development of regenerative medicine. Remarkably, in the early part of the new century, Shinya Yamanaka and colleagues found that nuclear reprogramming of mouse fibroblasts could be accomplished by the ectopic expression of four transcription factors (OCT4, SOX2, KLF4 and MYC, as OSKM), known as iPSCs [116] (Fig. 3b). Human iPSCs were established within a year by overexpression of the same combination of these four factors or different but overlapping factors [38, 37]. Intriguingly, retroviral transgenes overexpressing these four factors only need to be present at the generation of iPSCs. Once these cells are established, the exogenous genes are silenced, and the endogenous genes of the four factors are activated [117]. Moreover, PSCs could also be induced from mouse somatic cells using a combination of several small-molecule compounds [118]. Most small molecules used to date that facilitate somatic cell reprogramming are capable to substitute three of the four master regulators, SKM. The identification of molecules that can compensate directly for Oct4 transduction has proved difficult, until the first successful reprogramming experiment using only six small-molecule compounds. Two of these compounds were found using phenotypic cellular screening, a promising approach for further optimization of reprogramming conditions. [119, 118]. Despite the recent progress, a barrier remains to rapid and reliable induction of iPSCs from somatic cells, limiting their use in clinical settings. Future studies should emphasize on the incorporation of well-validated chemical probes and more sophisticated pharmacological approaches to discover optimal reprogramming conditions. In addition, further elaboration is required for a complete profile of how programing is initiated and subsequently consolidated.

### Trans-differentiation

Yamanaka’s induced reprogramming also highlights a new type of trans-differentiation approach known as direct reprogramming, whereby terminally differentiated cells could convert to other cell types. Transdifferentiation of pancreatic exocrine cells into insulin-producing β-cells has been described and was accomplished via forced expression of a series of transcription factors [120]. In addition, massive β-cell loss in the pancreas evoked some α-cells to transdifferentiate into β-like cells, in which alteration of cellular identity proceeded as a natural response to injury [121] (Fig. 3c). Naturally occurring transdifferentiation could also be perfectly illustrated in lens regeneration in newt. Once the lens is removed, the epithelial cells of the dorsal iris can undergo transdifferentiation and regenerate the missing lens [122]. Natural transdifferentiation occurs in two steps: first, the cell dedifferentiates and generates precursor cells. Then, the natural developmental program begins, directing the cell to generate into the new lineage [123]. However, it has been found through experimental induction of transdifferentiation that one cell type directly converts into another; in some cases, cells pass through an unnatural intermediate phase (Fig. 3d). Graf and colleagues found that monocytes, B cells, myeloid cells, and erythroid cells can convert into one another without undergoing an intermediate multipotent state within the blood lineage [124, 125]. Transdifferentiation offers new strategies for regenerative medicine, for example, fibroblasts in the heart can transdifferentiate and thus replace damaged cardiomyocytes.

### Stem cell plasticity in hair follicle

Tissue regeneration in mammals is more limited than in amphibians and invertebrates. Nevertheless, recent studies found that mammalian aSCs could display remarkable plasticity and reversibility, such as SCs in hair follicles, intestines, and the lung airway. The bulge of telogen hair follicles maintains heterogeneous and hierarchical SC compartments. The SCs located at the upper and lower bulges express CD34, whereas Lgr5-expressing SCs are restricted to the lower bulge and the hair germ [126]. During anagen, Lgr5+ SCs undergo more divisions than upper bulge cells [24] and give rise to the rapidly and asymmetrically dividing cells in the matrix that generate hair shaft [66]. At the end of hair cycling, the progeny of Lgr5+ SCs repopulate the CD34+ SC compartment in the upper bulge [24]. However, the Lgr5+ SCs in the hair germ do not contribute to another hair cycle. Instead, bulge cells constitute an alternative source of SCs that can give rise to new SCs in hair germ for the next cycle [66]. Although the bulge normally gives rise to hair germ SCs, hair germ can replenish an empty bulge niche following bulge cell ablation [127]. Furthermore, killed Lgr5+ HFSCs could be restored by CD34+ bulge SCs [128] (Fig. 3e). These studies underscore the close relationship between Lgr5+ hair germ SCs and CD34+ bulge SCs and their remarkable capacity to interconvert under certain circumstances.

### Intestine stem cell plasticity

The intestinal epithelium’s rapid self-renewing and turnover tissue in mammals is fueled by daily symmetric division of SCs in crypt bottoms to produce rapidly dividing daughter cells. These cells will further differentiate into secretory cells or absorptive enterocytes [129]. Animal models inducing apoptosis in proliferative cells of crypt units via irradiation and cytotoxic damage have proved a rapid restoration of crypt units, indicating that plasticity of nonproliferative or rarely dividing cells contributes to the regenerative process [130]. Consistently, crypt epithelial homeostasis is not perturbed by genetic ablation of Lgr5+ SCs, which could be compensated by the Bmi1+ SC population [17]. In addition, Dll1+ cells neighboring the SCs that are normally committed to the secretory lineage could convert to an ISC state after crypt damage [18]. Similarly, other quiescent cells in the crypt, which are committed precursors of Paneth cells and the enteroendocrine lineage during homeostasis, can give rise to all the major epithelium cell types following intestinal injury [131] (Fig. 3f). Thus, Lgr5+ ISCs are required for maintaining crypt integrity in a stable state, while under conditions in which the intestinal epithelium is severely damaged, such as gamma irradiation, context-dependent cellular plasticity enables the replenishment of ISCs and restoration of crypt.

### Cellular plasticity in trachea

In the mouse trachea, keratin 5 (K5)-expressing epithelial basal SCs are responsible for cellular replenishment. Basal cells have the potential to self-renew and give rise to Clara cells and ciliated cells during homeostasis and after injury, rendering basal SCs at the top of the cellular hierarchy in the tracheal epithelium [132]. Intriguingly, ablation of basal SCs results in the proliferation of differentiated secretory cells. Moreover, lineage tracing indicates that committed secretory cells convert to basal SCs. The converted cells were indistinguishable from SCs in phenotypical characteristics, and they could take part in repairing epithelial injury similarly to their endogenous counterparts [133] (Fig. 3g). Further ex vivo assays showed that secretory cells can dedifferentiate into basal SCs when basal cells are not present. However, direct cell contact of secretory cells with basal SCs is sufficient to inhibit secretory cell dedifferentiation [133]. The capacity of basal cells to inhibit the dedifferentiation of secretory cells, even with a single basal cell, exhibits many implications for general tissue biology, in which SCs and their progeny can interact with each other to modulate their relative ratios in tissue, and ensures a precise orchestration of epithelium architecture.

### Stem cells in EMT

The plastic transition between the epithelium and mesenchyme is called epithelial–mesenchymal transition (EMT) and is integral to normal development and cancer progression, in which epithelial cells acquire mesenchymal properties and exhibit decreased intercellular adhesion, enhanced motility and resistance to apoptosis [134]. EMT and cancer stem cell CSCs formation are two fundamental and well-studied processes that contribute to cancer metastasis and tumor relapse. EMT has been shown to modulate ES cell differentiation, induce reprogramming and CSC behavior. Correspondingly, the pluripotent ESCs in the inner mass of the blastocyst have epithelial features [135]. As these pluripotent epithelial epiblast cells form, the primary mesoderm proceeds through EMT during gastrulation. Thus, EMT is an initial differentiation event in the formation of three germ layers from PSCs [136]. In contrast, the reprogramming of fibroblasts into iPSCs represents the transition of a mesenchymal phenotype into an epithelial phenotype [137]. Moreover, induction of an EMT in immortalized human mammary epithelial cells results in the expression of mammary epithelial SC markers. Furthermore, these cells exhibit an increased capacity to form mammospheres, a property of mammary epithelial SCs [138]. Consistently, differentiated cancer cells could convert to CSCs through EMT, endowing oncogenic mutations in differentiated cancer cells to integrate into CSCs [139]. This scenario capacitates CSCs with updated oncogenic mutations to clonally expand and disseminate in evolutionary tumors [140]. Accumulating studies support that neoplastic cells within individual carcinomas often exhibit considerable phenotypic heterogeneity in their epithelial versus mesenchyma-like cell states, in which the cells can undergo a partial EMT to attain a hybrid epithelial/mesenchymal (E/M) phenotype or a complete EMT to attain a mesenchymal one [141–143]. And the plasticity in cell states is regulated by signaling pathways such as Notch. For example, certain breast cancer cells can reside stably in a highly tumorigenic, hybrid E/M state driven by Snail and canonical Wnt signaling [144, 145]. Moreover, these hybrid E/M breast cancer cells had a combination of several stem-like traits since they displayed increased plasticity, self-renewal and mammosphere formation [146, 145]. And the acquisition of a hybrid E/M state is essential for tumorigenicity of basal breast cancer cells [145]. Interestingly, the cells with hybrid or intermediate E/M state can shift towards either end (i.e., E or M) of the EMT spectrum, creating a window of opportunity for stemness (Stemness window). The size of the window is controlled by three input signals: NF-κB signaling level, EMT induction, or Notch activation. With different combinations of the three signals, various possible combinations of different types of cells can exist. For instance, a strong EMT induction narrows the window while a NF-κB activation can enlarge the window. A small window implies limited opportunities for stemness, result in the entire EMT space containing only epithelial cells or/and mesenchymal cells [147, 148]. And Jagged-Delta asymmetry in Notch signaling can result in a Sender/Receiver hybrid phenotype [149, 150]. In addition, the gene-expression profiles of mesenchymal-like and epithelial-like breast cancer stem cells resemble those of distinct basal and luminal stem cells found in the normal breast. And the plasticity of breast cancer stem cells might endow these cells with the capacity for tissue invasion, dissemination, and growth at metastatic sites [143]. Simulation study showed that the more mesenchymal CSCs lie at the invasive edge, while the hybrid epithelial/mesenchymal CSCs reside in the tumor interior [150]. These findings suggest that the design of future therapeutic strategies will need to consider the different subpopulations of carcinoma cells that reside at various positions along the epithelial–mesenchymal spectrum.

Thus, EMT confers SC traits to carcinoma cells that are associated with high-grade self-renewal, malignancy and resistance to apoptosis, which are dangerous for cancer patients. Conclusively, EMT programs are associated with the acquisition of SC characteristics in both normal and neoplastic cells (Fig. 3h). However, the relationship between EMT and SCs is largely unknown, which is why epithelial SCs always express a wide array of markers of mesenchymal cells. Intriguingly, a study showed the generation of iPSCs from mouse fibroblasts requires a mesenchymal-to-epithelial transition (MET) regulated by suppressing pro-EMT signals from the culture stimulus and promoting an epithelial program within the cells. Specifically, Sox2/Oct4 suppress the Snail (EMT mediator), cMyc inhibit the expression of TGF-β1 and TGF-β receptor 2, and Klf4 induces epithelial genes including E-cadherin. Consistently, preventing EMT in epithelial cells cultured with serum can generate iPSCs without cMyc and Klf4 [151]. This study indicates that MET as a key cellular mechanism toward induced pluripotency.

### Plasticity of CSCs

In addition to cellular plasticity in homeostasis and repair, studies in cancer biology found that CSCs and non-CSCs could undergo phenotypic transitions with certain stimuli. Breast cancer cell lines consist of cell populations that display SC-, basal-, or luminal-like phenotypes. All three subpopulations were capable of generating cells of the other two phenotypes [152]. Thus, CSC and non-CSC identities are plastic in this case. Recently, CSC plasticity has been further evaluated in xenografted human cancer models. An inducible version of the suicide gene caspase 9 was used to delete Lgr5+ CSCs in human colorectal cancer xenografts. Additionally, deletion of Lgr5+ CSCs reduced tumor size, while removal of the apoptosis inducer resulted in the regrowth of tumors. Additionally, the proliferation of KRT20+ and differentiated tumor cells occurred simultaneously with tumor regeneration. Further assays via lineage tracing of the differentiated tumor cells demonstrated that these cells restored the LGR5+ CSC pool [153] (Fig. 3i). In contrast, the hierarchy of glioblastoma appears to be unidirectional and irreversible. The ablation of CSCs ceased tumor growth without CSC regeneration from other glioblastoma cells [154]. Consistently, several transcription factors are essential for the maintenance of glioblastoma CSC identity. Once these transcription factors are re-expressed, differentiated glioblastoma cells can be converted into CSCs with fully tumorigenic capacity [155]. In conclusion, CSC hierarchies are not rigid but rather plastic, and non-CSCs reprogramming to CSCs might be a common phenomenon that is induced by various environmental stimuli. Thus, the plasticity of CSCs might be a potential therapeutic target in some human cancers.

## Epigenetic regulation of stem cells

The embryonic development of mammals is a tightly regulated process in which distinct cell types are generated in a highly ordered manner, and this process is established by tight transcriptional and epigenetic manipulation. Epigenetics is a stably heritable yet reversible phenotype of alteration in a chromosome without changes in the DNA sequence [156]. DNA methylation is a major epigenetic modification that plays a crucial role in transcriptional suppression. In addition, chromatin is subject to a diverse array of posttranslational modifications at the histone tails. DNA methylation and histone modifications and their crosstalk affect the activity of specific regulatory elements, such as promoters and enhancers. Promoters are the platform on which the transcription factors and RNA polymerase II (RNAPII) assemble during the initiation of transcription. Enhancers are typically 200–300 bp in length and defined based on an assay of histone modifications and proximity to the gene body [157].

### Epigenetic traits during stem cell differentiation

**Fig. 4:**
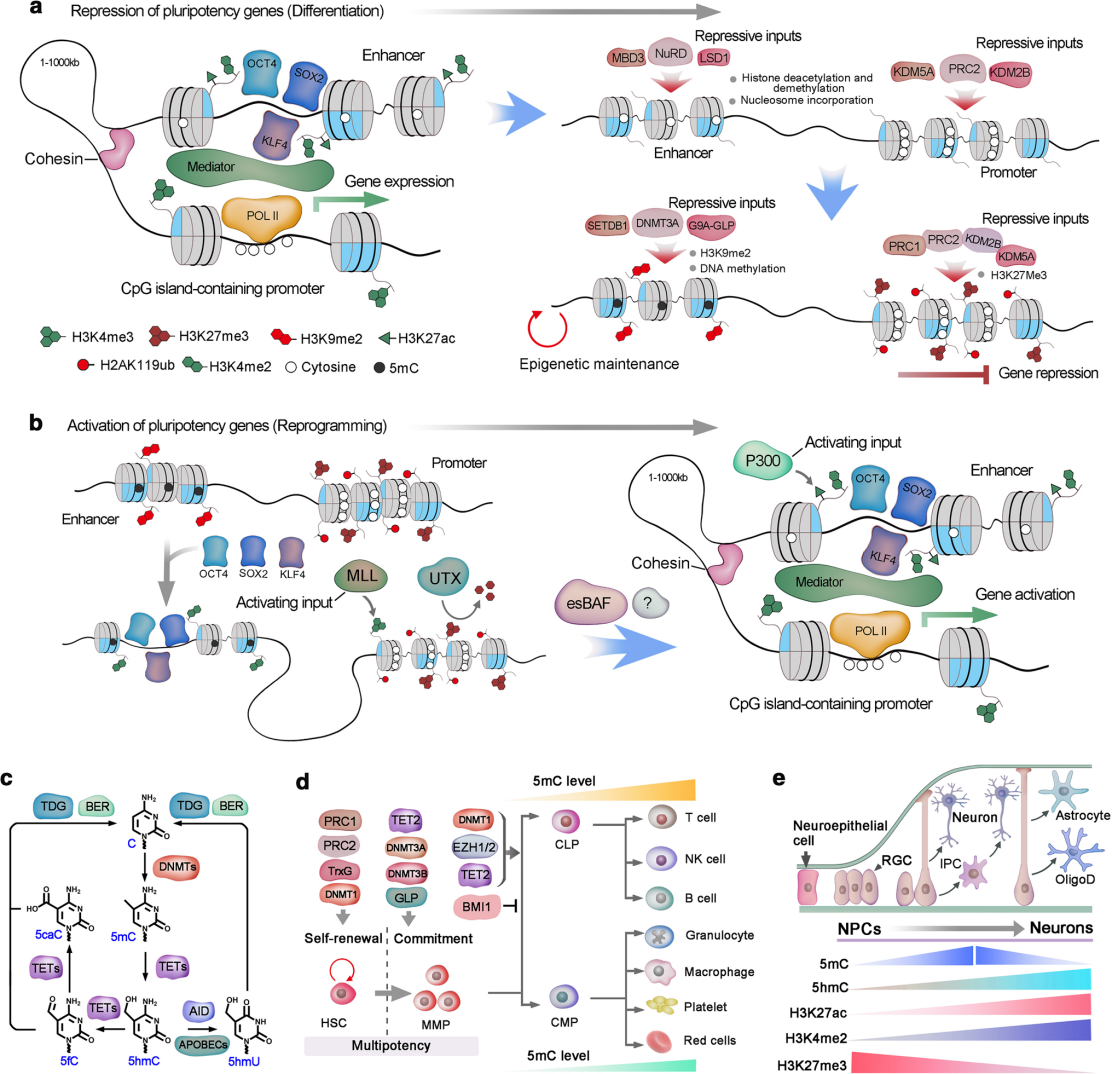
Epigenetic regulation of stem cells. **a** Active regulatory elements are typically enriched for 5hmC, H3K27ac, H3K4me, and bound Mediator complex. During gene repression, the activating histone modifications are eliminated, and repressive marks, such as H3K27me3 and nucleosomal compaction, are established. In pluripotent stem cells, multiple enhancers combined with master pluripotency transcription factors, such as OCT4, SOX2 and KLF4, establish a super-enhancer, which supports the activation of pluripotency genes. The absence of master pluripotency factors could induce the disassembly of the enhancer–promoter complex in the assembly of repressive inputs. The nucleosome remodeling and deacetylase (NuRD) complex induces nucleosome formation at the binding region of pluripotency factors and alters the histone modifications that correspond to transcriptional activity. Similar disassembly of activating modifications occurs in the promoter by PRC2-associated histone demethylases KDM2B and KDM5A. Furthermore, H3K9me2 or H3K9me3 is deposited in the enhancer by G9A in complex with GLP and SETDB1, and DNA methylation by DNMT3A. Equally, PRC2 deposits H3K27me3 in promoters, which initiate chromatin compaction by recruiting the canonical PRC1 complex and monoubiquitylate H2AK119 (H2AK119ub). K27ac, Lys27 acetylation; Pol II, RNA polymerase II. PRC2, Polycomb repressive complex 2. GLP, G9A-like protein. DNMT3A, DNA methyltransferase 3A. CCTC-binding factor (CTCF). DNMT, DNA methyltransferase; HDAC, histone deacetylase; HAT, histone acetyltransferase; MED, mediator complex; MBD, methyl-DNA-binding domain protein; POL II, RNA polymerase II; TET, ten eleven translocation dioxygenase; TrxG, trithorax group complex. TF, transcription factor. **b** In differentiated cells, H3K27me3 and H2AK119ub are enriched in CpG island-containing promoters of some pluripotency genes. At the initiation of reprogramming, OCT4, SOX2 and KLF4 (known as OSK) bind to partial motif sequences of some select enhancers to engage pioneer factor-like activity. These OSK binding sites are modified with H3K4me1 and H3K4me2. OSK binding also promotes H3K4 methylation at the promoter via the MLL component WDR5 and is associated with local erasure of H3K27me3 by the histone demethylase UTX. Subsequently, OSK cooperates with unknown factors to establish a canonical enhancer architecture, including H3K4me2 and H3K27ac, and stable topological cognation to the promoter via the cohesin and Mediator complexes. Embryonic stem cell-specific BAF (esBAF) further stabilizes the enhancer-promoter complex, and the activating inputs predominate at this stage and direct the expression of the target genes. LSD1, Lys-specific demethylase 1; MBD3, methyl CpG-binding domain protein 3; MLL, mixed lineage leukemia. Pol II, RNA polymerase II, WDR5, WD repeat-containing protein 5. **c** The mechanisms of DNA methylation and demethylation. Cytosine is converted to 5mC by DNMTs. TET1 is responsible for the conversion of 5mC to 5hmC. Three TET family proteins could subsequently oxidize 5hmC to 5fC (5-formylcytosine) and then to 5caC (5-carboxylcytosine). Moreover, 5hmC can be converted to 5hmU (5-hydroxymethyluracil) by deaminase activation-induced cytidine deaminase (AID) and apolipoprotein B mRNA-editing enzyme catalytic polypeptides (APOBECs). 5fC, 5caC and 5hmU can be excised by thymine DNA glycosylase and replaced by an unmodified cytosine via the base-excision repair (BER) pathway. **d** Key epigenetic regulators that are involved in HSC self-renewal and lineage commitment during differentiation, and DNA methylation levels changes during hematopoietic lineage commitment. CLP, common lymphoid progenitor; CMP, common myeloid progenitor; DNMT, DNA methyltransferase; DNA me, DNA methylation; GLP, G9A-like protein. GMP, granulocyte–monocyte progenitor; MPP, multipotent progenitor; PRC, polycomb repressive complex; TET, Ten-eleven translocation; TrxG, Trithorax group. **e** In the embryonic neurogenesis of mice, neuroepithelial cells develop into radial glial cells (RGCs) around embryonic day 14. RGCs can either produce neurons directly or give rise to intermediate progenitor cells (IPCs), which in turn generate neurons. During later embryonic development, RGCs also give rise to oligodendrocytes (OligoD) and astrocytes

Epigenetic regulation is highly dynamic during the differentiation of mouse PSCs and human PSCs (hPSCs), especially at the initial transition from the pluripotency state to a lineage-committed cell [158]. In PSCs, the activity of master pluripotency factors such as OCT4, SOX2, and NANOG is focused on the super-enhancers of the target genes, with the assistant of the mediator and cohesin complexes, to control target gene expression [159]. However, the super-enhancer complex is precisely sensitive to cellular state, and interruption of mediator activity or attenuation of master pluripotency factors results in dramatic downregulation of the target genes [160]. With the onset of PSC differentiation, the pluripotency-associated enhancer architecture is disassembled in a stepwise manner, initiated with the removal of histone modifications and encroachment of nucleosomes, and followed by culmination of the methylation at DNA- and repression-associated histone residues [161]. The nucleosome remodeling and deacetylase (NuRD) complex, including LSD1 and methyl CpG-binding domain protein 3 (MBD3), counteract the activity of histone acetyltransferases and leads to the disassembly of pluripotency-associated enhancers [162, 163, 161]. Loss of histone acetylation and methylation modifications, associated with transcriptional activity in enhancers, results in a similar decommissioning of promoter-associated activating modifications [164] (Fig. 4a). Simultaneously, the enhancers and promoters of the pluripotency state are shut down, and chromatin is compacted.

### Activation of silent pluripotency genes of differentiated cells

Transcription factors tend to bind within open chromatin. However, pioneering factors can directly bind to related DNA motifs, evict nucleosomes and initiate enhancer activation, even in compact chromatin [165]. Thus, the introduction of a minimal set of pioneer factors into a somatic nuclear environment, in which the majority of the pioneer factors (OSKM) target loci are repressed, initiates preliminary epigenetic remodeling and induces nuclear reprogramming [166, 167]. In the context of reprogramming, OSKM cooperatively bind to nucleosomal DNA without obvious histone modifications and maintains recognition motifs for these pioneer factors [168]. However, similar loci with repressive epigenetic modifications tend to be intransigent to the binding of pioneer factors [168]. Moreover, unlike canonical pioneer factors, OSKM lack a DNA-binding domain that could outcompete nucleosomes to establish a region of open chromatin, and OCT4, SOX2, and KLF4 (OSK) seem to cooperatively bind to outwardly facing partial motif sequences of their shared somatic targets within the nucleosome [169]. The initial binding of OSK within somatic cell chromatin appears to be the earliest step in initiating the pluripotency network; however, the extended latency between OSK binding and the induction of pluripotency genes suggests that these primary interactions are insufficient [170]. By contrast, cMyc is unique among the reprogramming factors, as it is neither a component of the core pluripotency network nor definitely necessary for reprogramming to iPSCs [171]. Even though cMyc is a crucial factor in many various biological processes, including cell growth and differentiation. And there are studies strongly support that cMyc is a nonlinear amplifier of transcriptional outputs that acts universally on active genes containing the E box DNA motif [172, 173]. Therefore, cMyc occupies the core promoter regions of many active genes in ESCs/iPSCs and is typically not present at enhancers [167]. Chromatin remodeling could be further achieved by additional regulators, such as cell-type specific BAF complexes, which evict nucleosomes around the loci of transcription factor binding and stabilize a nucleosome-depleted site [170, 165]. Consistently, aberrant expression of the ESC-specific BAF (esBAF) complex components during reprogramming promotes iPSC generation [174]. Moreover, primary genomic binding of OSK in somatic cells seems to depend on corresponding chromatin status, such as OCT4 preferentially binding at distal *cis*-regulatory sequences that lack DNA methylation but are nucleosomal [175]. The binding of OCT4 initiates chromatin modification at the enhancer, which interacts with cognate CpG island-containing promoters to induce targeted deposition of H3K4 methylation and H3K27 demethylation [176, 177]. Thus, the preliminary enhancer activation must be followed by the assembly of coregulators to eventually induce the activation of target genes (Fig. 4b). Additional chromatin regulators that are essential for the process have been found. For example, the H3K27me demethylase Utx also interacts with OSK and is important for the deletion of this repressive H3K37me3 from pluripotency loci. While these additional regulatory factors need to function collaboratively with OSKM for binding to repressed pluripotency genes, such an opportunity may arise during normal cell division, immediately following DNA replication before nucleosome assembly. It is still elusive whether replication is essential for changing gene expression patterns at every stage of the reprogramming process [178].

Together, these chromatin dynamics are likely crucial for the turn-off of the somatic expression program and the transition to pluripotency. The exact nature of the interactions among these coregulators is still unclear, although current studies have highlighted a number of crucial players that direct the reprogramming process. However, with manipulation of several pioneer factors, we could generate iPSCs from somatic cells.

### Epigenetic regulation in hematopoiesis

HSCs give rise to the highly specialized cell types in the mammalian blood system [9]. Accordingly, the cell types of hematopoietic systems can be distinguished based on their differential epigenetic modification [179, 180]. Likewise, genes that are subject to demethylation and transcriptional activation during lineage commitment always have a functional role that is specific to the corresponding lineage [181]. Differentiation of HSCs toward the myeloid lineage showed less global DNA methylation than commitment toward the lymphoid lineage. This suggests that myeloid commitment may be a ‘default’ state of HSCs, whereas HSC commitment along the lymphoid lineage requires proper DNA methylation, which could repress myeloid lineage-defining genes [182]. DNA methyltransferases (DNMTs) catalyze the transfer of a methyl group to carbon 5 of the cytosine ring to form 5-methylcytosine (5mC), which can be hydroxylated to 5-hydroxymethylcytosine (5hmC) by ten-eleven translocation (TET) dioxygenases [183] (Fig. 4c). DNMT1 plays an important role in HSC maintenance and lymphoid lineage commitment, whereas DNMT3A and DNMT3B are indispensable for HSCs to exit the multipotent state [184, 185]. TET2 oxidizes 5mC to 5hmC and is highly expressed in HSCs, and deletion of TET2 increases the level of 5mC. However, the phenotype of TET2 ablation in HSCs resembles a combinative phenotype of DNMT1 loss and DNMT3A and DNMT3B deficiency [186]. Therefore, DNA methylation and hydroxymethylation intricately interact to modulate proper gene expression and cellular function (Fig. 4d). Thus, predicting phenotypic outcomes based on the global state of methylation and hydroxymethylation is insufficient. Instead, DNA methylation of unique loci specifically affects HSC function. Additionally, the expression of key components of polycomb repressive complexes (PRCs) is associated with the status of HSC differentiation. For example, BMI1, a component of PRC1 that monoubiquitylates H2AK119, is highly expressed in HSCs [187]. The loss of BMI1 leads to progressive hematopoietic failure by inducing HSC cell cycle arrest, apoptosis, premature differentiation and defective self-renewal [188, 189]. In contrast, ectopic BMI1 expression promotes HSC self-renewal and expansion of the HSC pool [190].

Histone acetylation favors transcriptional activation by leading to the loosening of chromatin. The histone acetyltransferase cofactor TRRAP promotes HSC self-renewal, and its ablation leads to BM failure [191]. Histone deacetylases (HDACs) HDAC1 and HDAC2 counterbalance the activity of histone acetylation (Fig. 4d). Likewise, a combined loss of HDAC1 and HDAC2 results in extensive hematopoietic failure with the phenotypes of severe anemia and cytopenias [192, 193]. Intriguingly, these phenotypes are similar to the characteristics of aged HSCs, which suggests that there might be an association between aged HSCs and deregulated histone acetylation and that aged HSCs could potentially be restored by HDAC inhibitors.

### Epigenetic regulation in ISCs

The cell fate of ISCs and non-SCs in the intestinal epithelium SC pool showed extensive plasticity [129]. Accumulating data indicate that epigenetic regulation plays a role in the plasticity of SC hierarchies within intestinal epithelium. Loss of HDAC enzymes (HDAC1/2) perturbs cell lineage commitment in the intestine [194]. Histone marks that permit chromatin accessibility surprisingly do not significantly differ between ISCs and their progeny cells, both of which showed prominent histone marks in many intergenic regulatory regions [195]. This implies the underlying epigenetic mechanism of ISC plasticity, namely, the regulatory regions of intestinal cells are continuously accessible to lineage-specifying transcription factors, niche factors and other environmental factors to alter cell fate via dedifferentiation or transdifferentiation. DNA methylation surrounding transcription start sites (TSS) typically correlates with gene repression [196]. Furthermore, DNA methylation is generally most dynamic at regulatory regions outside the TSS, although the functional significance of these dynamics is often unclear. DNA methylation was found to be static at TSS during ISC differentiation, and minimal changes were detected mainly at enhancer elements [197]. However, deletion of Dnmt1, an enzyme that maintains DNA methylation, led to crypt expansion, indicating the crucial role of global DNA methylation in ISC differentiation [197]. Given that Dnmt3b knockout, which leads to loss of de novo methylation, exhibits a negligible effect on intestine homeostasis [198], the functional importance of these minimal local DNA methylation changes during differentiation require further investigation. Moreover, additional studies are needed to explore the role of other epigenetic factors, such as chromatin remodelers, in intestinal cell plasticity.

### Epigenetic mechanisms in neurogenesis

Neurogenesis is the process in which NSCs or neuronal progenitor cells (NPCs) generate new neurons [199]. In mouse embryonic neurogenesis, neuroepithelial cells are transformed into radial glial cells (RGCs) in the ventricular zone (VZ) and SVZ [200]. Initial RGCs function as fate-restricted NPCs that either directly generate nascent neurons or produce neuronal intermediate progenitor cells (IPCs), which in turn give rise to neurons [200, 201]. During later development, RGCs also give rise to astrocytes and oligodendrocytes [200]. Even though most RGCs terminally differentiate into mature neural cells, a small portion of RGCs remain quiescent during embryonic development, and these preserved RGCs eventually become aSCs that are responsible for SVZ neurogenesis [202]. Many intrinsic signals, such as rapid epigenetic changes, work synergistically to support robust embryonic neurogenesis [203]. As DNMTs are the primary writers of DNA methylation, proper function of DNMTs is crucial for neurogenesis. DNMTs exhibit dynamic spatial and temporal expression during neurogenesis, during which DNMT1 is extensively detected in the ventricular neurogenic layer in the embryonic mouse brain and responsible for maintaining DNA methylation status during rapid cell replication [204]. DNMT3B is robustly expressed in the VZ between E13.5 and is undetectable after E15.5. In contrast, DNMT3A is initially detected primarily in NPCs within the VZ and SVZ from E10.5 to E17.5 and is constantly expressed in postnatal neurons [205]. Deletion of Dnmt1 and Dnmt3b in ESCs leads to embryonic lethality [206]. In addition, genome-wide analyses of DNMT3A-mediated site-specific DNA methylation in embryonic NPCs have uncovered its direct epigenetic regulation in many neurogenic genes [207]. DNA methylation can be ‘passively diluted’ during cell replication, and TET1 was only recently found to catalyze the conversion of 5mC to 5hmC. Additionally, 5mC can be converted into unmodified cytosine by TET family proteins and other proteins [208] (Fig. 4e). TET proteins are the major players in DNA demethylation and play crucial roles in neurogenesis. Tet1 expression is higher in mESCs than in NPCs; however, Tet2 expression is comparable between ESCs and NPCs, while Tet3 is upregulated in NPCs and expressed minimally in mESCs [209]. Tet1 knockout mice function in embryonic and postnatal development and show normal morphology [186]; however, *Tet1*−/− adult mice exhibit a decreased number of NPCs in the SGZ and specific impairments in extinction learning and short-term memory [210, 211]. Tet3 is important in early embryonic development; Tet3 deletion results in embryos that either arrest and do not survive or mice that survive embryonic development but die perinatally for unknown reasons, partially because Tet3 deletion impairs the expression of key epigenetic reprogramming genes, such as Oct4 and Nanog [212].

Histone methylation/demethylation is precisely orchestrated to ensure expression of the correct set of genes at different neurogenic stages [213]. During neocortical development, the polycomb group (PcG), which is responsible for producing active histone modifications such as H3K4me3, plays important roles in the NSC neurogenic-to-astrogenic transition. Consistently, depletion of PcG components, such as Ring1b or Ezh2, leads to an extended neurogenic phase and delayed onset of astrogenesis during neocortical development of embryonic mice [214]. Lysine-specific histone demethylase 1 (LSD1) was the first identified histone lysine demethylase and selectively demethylates H3K4me2 and H3K4me1, and knockdown of LSD1 extensively attenuates NPC proliferation in the DG of adult mice [215]. Similar to histone methylation, histone acetylation is a reversible process that is triggered by HDACs. More than 18 HDACs have been found to regulate histone deacetylation in the mammalian genome, and some HDACs are involved in neurogenesis. For example, the expression of HDAC2 increases during the differentiation of NSCs into neurons, whereas HDAC1 is detected at high levels in glial cells in the adult brain [216]. Many small-molecule HDAC inhibitors have been developed to manipulate histone acetylation, including valproic acid, which promotes NPC differentiation in the adult hippocampus by regulating the expression of a neurogenic differentiation factor [217]. However, despite the impressive progress that has been made, a more comprehensive picture of the participation of HDACs in neurogenesis is still desired and requires further investigation.

### Epigenetics in pancreatic cancer stem cells

Pancreatic Cancer (PDA) is an aggressive malignancy characterized by early metastasis and a high mortality. using lineage tracing markers or by assessment of tumorigenesis in xenograft, CSCs in PDA were empirically defined [39, 218]. These CSCs and their progeny also exhibit a significantly altered epigenetic profile with distinctive patterns of DNA methylation, and the CSCs of PDA showed higher overall DNA methylation levels than the remaining cancer cells. DNMT1 inhibition results in dose-dependent reduction of the CSCs population and reduced self-renewal marker, and the CSCs showed increased commitment and epithelial-like differentiation [219, 220]. Thus, efforts to disrupt DNA methylation should form a part of our therapeutic strategy to destroy the pancreatic CSC compartment. In addition, several preliminary studies in established pancreatic cancer cell lines support that interference with histone modifications can obviously inhibit the CSC proliferation and potentially synergize with existing chemotherapy to inhibit pancreatic tumor growth [221, 222]. In addition, several mechanisms of interaction between epigenetic and metabolic pathways in PDA were found and which ultimately result in the observed cellular plasticity and enhanced tumorigenesis [223]. An example of metabolic and epigenetic crosstalk is the alterations in metabolism pathway that produces high levels of glycolysis and serine biosynthesis in PDA consequently led to generation of large amounts of *S*-adenosylmethionine, which in turn promotes hypermethylation in specific retrotransposon elements and associated with transcriptional silencing [224]. In the future study, it will be important to elaborate these findings with more high-fidelity models of pancreatic cancer, such as primary patient-derived xenografts and tumor organoids.

## Stem cell application and perspectives

SCs, which build all the structural and functional units in the human body, are promising treatments for many incurable diseases, such as hematological disorders, cardiac disease and Alzheimer’s disease. Among the SCs that present at various stages of life (embryonic, fetal, and adult), aSCs exhibit great potential and are the safest for clinical application.

### HSCs in clinical applications

**Fig. 5:**
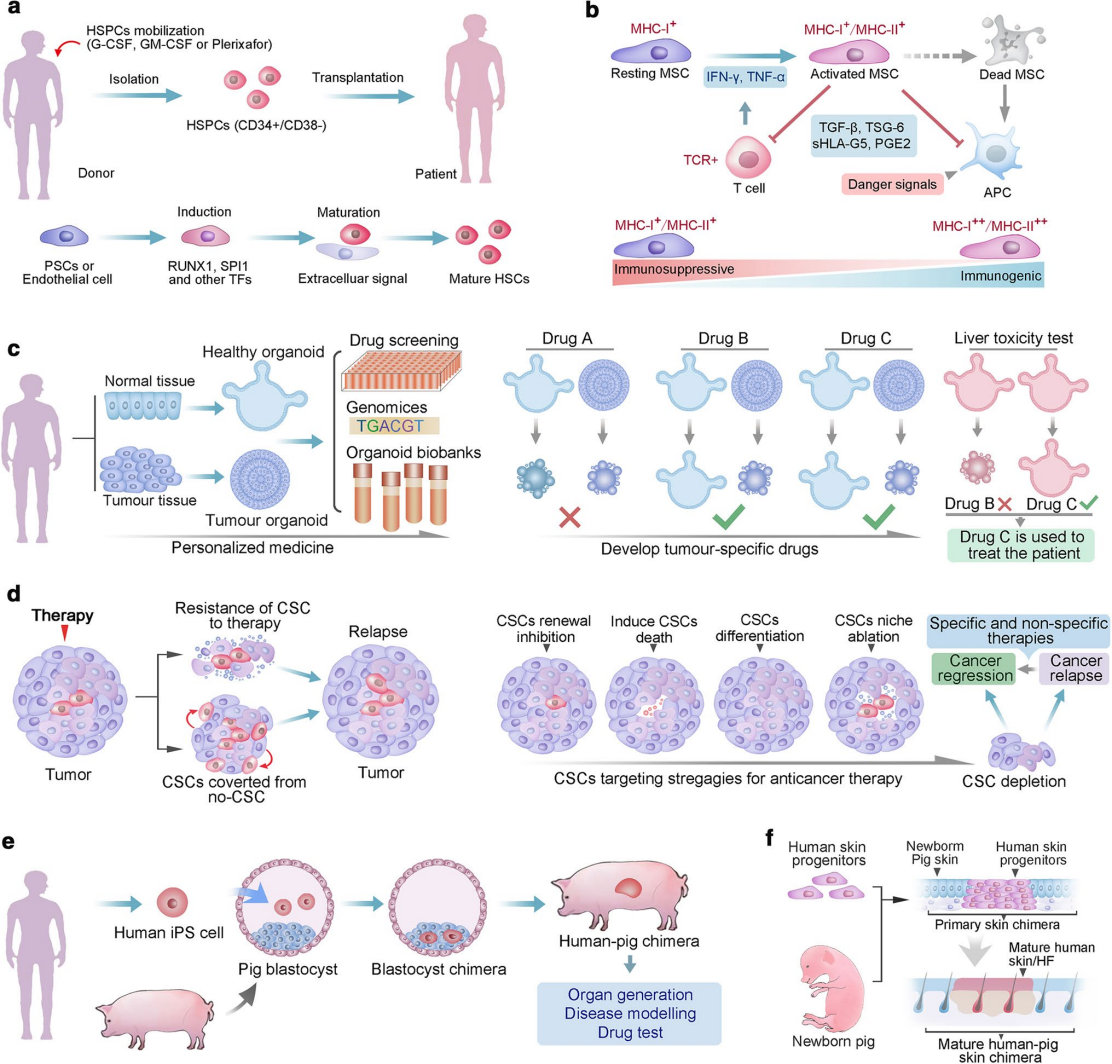
Stem cell application and perspectives. **a** Two distinct procedures to collect HSCs for transplantation. HSCs are isolated from donor blood cells, in which the HSCs are mobilized with G-CSF, GM-SCF or plerixafor, and enriched with HSCs marker of CD34+/CD38−. Alternatively, protocols have established to produce HSCs ether from endothelial cells, or from human pluripotent stem cells (PSCs), these two protocols treated initiate cells with overlapping cocktails of transcription factors. The primary HSCs need to receive as-yet-unknown extracellular signals for further maturation. **b** MSC immunosuppressive capacity and immunogenicity are affected by levels of systemic or local inflammatory cytokines. High immunosuppressive potential of MSCs is achieved via suppression of T cell activation and inhibition of antigen-presenting cell (APC) maturation. Whereas, MSCs that do not tip the balance toward immunosuppression are prone to immunogenicity and result in immune detection and destruction, as debris from apoptotic MSCs are processed by APCs in the context of danger signals. The rate of immune detection of allogeneic MSCs is determined by the balance between relative expression of immunogenic and immunosuppressive factors in MSCs. IFN-γ, interferon gamma; MHC, major histocompatibility complex; PGE2, prostaglandin E2; sHLA-G5, soluble human leukocyte antigen-g5; TCR, T cell receptor; TGF-β, transforming growth factor beta; TNF-α, tumor necrosis factor alpha; TSG-6; TNF-stimulated gene 6 protein. **c** Organoids generated from patient-derived healthy and tumor tissues can be genetically characterized and used for drug screening, and can be cryopreserved and stored in living organoid biobanks. Organoids developed from healthy tissue of the same patient can be used to screen drugs that are less toxic to healthy cells while selectively kill tumor cells. Moreover, hepatocyte organoid cultures may be used to test for hepatotoxicity. In this schematic example, drug C could specifically kills tumor organoids and does not show hepatotoxicity, and thus it seems most suitable for treating the patient. **d** CSC model of cancer relapse. Intrinsic and extrinsic mechanism contribute to the CSCs resistance to the medical therapy, in addition, non-CSCs may convert to CSCs and replenish the CSCs pool, ether CSCs drug resistance or replenish result in cancer relapse. The CSC model suggests that inhibiting CSC self-renewal, inducing CSC specific cell death, inducing CSC differentiation or targeting CSC niche would lead to the depletion of the CSCs pool and subsequent tumor regression. Nevertheless, if the CSC is reversed from no-CSCs, further specific and no-specific therapies will be needed the for the final regression of tumor. **e** The principle of interspecies blastocyst complementation for the generation of human–animal chimaeras. Human PSC-derived organ could help to solve the severe shortage of organ donors. Additionally, Human–animal chimaeras could be useful for modeling human diseases and for testing the efficacy and safety of a candidate drug in vivo. **f** The principle of tissue complementation chimera. In this example, human-pig integumentary chimera was achieved via transplanting human skin progenitors to the skin incision of newborn pig. The engraftment of human progenitors will develop to mature human skin tissue with appendage organs, such as hair follicle

BM transplantation is widely applied in SC-based therapy for the treatment of malignant and nonmalignant hematological disease. Edward Donnall Thomas performed the first human BM transplant in the 1950s [225]. HSCs, derived from BM as well as from mobilized peripheral blood (MPB) and umbilical cord blood (UCB), have been used for the treatment of hematological disorders, hemoglobinopathies, immune system disorders, myeloproliferative disorders and inherited metabolic disorders [226]. Additionally, by supplying hematopoietic transcription factors, hematopoietic cells can be generated from cells as diverse as fibroblasts, endothelial cells, and differentiated blood cells [227–229]. Moreover, PSCs, including ESCs and iPSCs, provide alternative sources for obtaining HSCs. However, generating functional human HSCs from PSCs is a challenge. Recently, George Daley and colleagues differentiated hPSCs to the hemogenic endothelium via a morphogen-based approach, and then seven transcription factors were identified to sufficiently convert hemogenic endothelium into hematopoietic SCs and progenitor cells that allow engraftment of myeloid, B and T cells in primary and secondary mouse recipients [230] (Fig. 5a). This approach holds promise for recapitulating hematopoietic disease in humanized mice and for therapeutic strategies to restore hematopoietic function in genetic blood disorders.

### MSCs in clinical trials

Mesenchymal stem cells (MSCs) are characterized by their regenerative properties and capacity for differentiation of multiple cell lineages and hold extensive promise in cell-based therapies for various diseases. MSCs were initially identified in 1967 by Friedenstein as fibroblast-like clonogenic cells from mouse BM and named colony-forming unit-fibroblasts (CFU-F); later, these cells were referred to as MSCs [231]. However, to date, a rigorous in vivo demonstration of MSC origins and phenotypes has not been established. Indeed, assays to identify and characterize MSCs are mostly based upon in vitro work, typically based on cell surface markers and multiple-lineage differentiation potential. Irrespective of these uncertainties, the clinical value of MSCs is achieved via their trophic and immunomodulatory properties, namely, MSCs produce extracellular vesicles, including exosomes, and a mass of cytokines and growth factors that inhibit immune responses (Fig. 5b) [232, 233]. Based on the immunomodulatory capacity of MSCs, the first clinical trial of MSCs was administered to a boy who suffered grade IV GvHD (graft versus host disease) after BM transplantation, and encouraging outcomes were observed in this case [234]. Since then, clinical trials using MSCs have soared. Today, more than 300 clinical trials of MSCs are underway investigating the treatment of various diseases, such as myocardial infarction, GvHD, amyotrophic lateral sclerosis (ALS) and diabetes. In 2011, MSC therapy for acute myocardial infarction gained approval in South Korea as the first MSC therapy with regulatory approval [235]. Whereas clinical studies of a small number of patients have shown MSCs with great clinical potential, hard evidence of a beneficial effect of MSCs from a large placebo-controlled trial remain elusive. Several placebo-controlled trials have provided disappointing results, with marginal or no benefit over placebo. To realize the potential of MSC therapy, MSCs should be optimized to extend their persistence and to avoid the production of alloreactive antibodies. Specifically, further studies should emphasize the elaboration of MSC characteristics in immunogenicity, potency and disease-specific mechanisms of action.

### Promising translational application of organoids

In addition to the potential of ISCs in regenerative medicine, SCs can give rise to organ-like structures known as organoids, which hold great promise for studying normal development and disease processes and open up new avenues for medical research and drug discovery. Disease research commonly involves animal models and experiments. However, profound differences in genetics, metabolism, size and life span all contribute to the fact that most pharmaceuticals developed in animals ultimately fail in human clinical trials. Organoids provide an alternative to animal-associated research strategies and exhibit human cell metabolism and homeostasis, and organoids have already been used successfully for the establishment of personalized human cancer models and for the assessment of patient-specific therapeutic efficacies of cystic fibrosis drugs (Fig. 5c) [236, 237]. Transplantation of organoids to repair damaged organs has been highlighted in liver SC-derived organoids, as liver organoids represent an available and lasting hepatocyte source; thus, organoids potentially provide revolutionized prospects for patients with liver disease [238, 239] The exploration of organoids was highlighted by the study in which intestinal crypt villus units can be built from a single stem cell in the absence of a non-epithelial cellular niche [91]. And organoids derived from a single Lgr5+ ISC can engraft onto injured intestinal mucosa and promote its recovery in a mouse IBD model [240]. In addition, long-term growth of primary kidney tubular epithelial organoids also has been established, which is valuable for personalized disease modeling [241]. Moreover, human pluripotent-stem-cell-derived intestinal tissues with a functional enteric nervous system have been established, which highlights the potential to develop organoids with more complicated cellular composition [242]. Despite the explosively developing field of stem cell-based organoids, current versions of organoids have clear limitations, e.g., innervation, blood vessels, and immune cells are still absent, thus disease processes are only partially recapitulated. In further investigations, more organoid-based regenerative therapies will be developed with improved culture and transplantation procedures, nontumorigenicity and economical expansion of organoids. Organoids remain genetically and phenotypically stable for a long time, which allows organoids to be used for a wide spectrum of applications in cancer research. Currently, large collections of patient-derived organoids of tumors and matched healthy tissue have been generated and biobanked (Fig. 5c). Recently, an organoid biobank of breast cancer tissues from > 100 patients was established. These organoids not only represent genetic and histopathological features of breast cancer but also maintain the expression of breast cancer biomarkers. Consistently, a proof-of-principle drug screen in these organoids with different drugs that target HER signaling showed that drug sensitivity status typically correlates with HER2 levels [243]. These data support that organoid biobanks have predictive value for drug efficacy in the treatment of individual patients. In addition, profiling of patient-derived organoids may uncover the underlying epigenetic and/or genetic mechanism of drug resistance, after which individual patients could be stratified for personalized cancer treatment. For example, a colorectal cancer organoid biobank was used to explore the efficacy of various RAS pathway inhibitors, in either a single or combinatorial manner, that have been used in the clinic [244]. Moreover, single SC-derived and long-term-cultured organoids were used to determine the genome-wide mutation patterns in distinct healthy SCs, and the related results suggest that tissue-specific mutagenic processes contribute to the accumulation of specific types of somatic mutations during malignant transformation [245]. Despite these advantages and applications, organoids also exhibit limitations. One of the intrinsic limitations is the lack of stroma, blood vessels and immune cells in cultured organoids, especially the immune cells in cancer organoid, as increasing evidence supports that much of tumor progression is intimately connected to its interaction with the immune system. Notably, a recent study employed iPSC-derived tissue engineering and integrated an intestinal organoid with functional neural cells [242]. Future studies should explore the possibility of establishing organoids with more complex structures.

### Target cancer stem cells

CSCs are characterized by their capacity to fuel tumor propagation and may contribute to tumor relapse and metastasis, and CSCs that are intrinsically or extrinsically instructed by the tumor microenvironment are more resistant to medical therapy than are ‘differentiated’ tumor cells [246]. An increasing number of studies have demonstrated that CSCs are enriched after chemo- or radiotherapy; for example, radiation therapy results in the enrichment of CSCs in xenografts GBM and breast tumors [247, 248]. In addition, CSCs appear to be resistant to DNA damage-induced cell death, similar to their counterparts in normal tissue [249, 250]. However, targeting CSCs necessitates a more comprehensive understanding of the mechanisms that support their resistance to therapies. CSCs have been shown to exhibit one or more aberrations in various signaling pathways, including Notch, Wnt and Hedgehog (HH) pathways, which are most likely crucial to the tumorigenicity of CSCs [251]. Therefore, targeting CSCs via regulation of the Wnt, HH and Notch signaling pathways holds the promise of inhibiting disease relapses. It is now clear that all signaling pathways function as a coordinated network [252]. The phenotype of CSCs is an output of the overall signaling network. Thus, the development of CSC-targeting agents should be based on a functional understanding of key nodes in the CSC signaling network. Consistently, effective antitumor activity was not detected by targeting CSCs with Notch, Wnt or HH inhibitors, either as single agents or in combination, in clinical trials. However, comprehensive approaches to modulate the interaction among Notch, HH and Wnt pathways, as well as other signaling pathways, showed promising antitumor effects in preclinical models [251].

If CSCs fuel tumor propagation, killing CSCs, targeting CSC proliferation or forcing CSCs to differentiate are alternative and independent approaches that potentially inhibit tumor growth (Fig. 5d). Practically, most patients with acute promyelocytic leukemia are treated with regimens that promote the differentiation of leukaemic cells [253]. The discovery of drugs that are able to inhibit CSC proliferation without assaulting the pool of normal SCs is the basic criterion for new anticancer therapies [254]. However, CSC-targeting therapies assume a rigid SC hierarchy, and therefore, the dedifferentiation of non-CSCs to CSCs could attenuate the clinical applications of such therapies. Indeed, using tumor organoid models, genetic lineage tracing and ablation systems, a recent study provided definite proof of functional plasticity within human CRC cells. The temporal effect of CSC targeting was eventually overwhelmed by the robust reversion of nontargeted cancer cells [153]. Thus, given the potential treatment relapse from non-CSC plasticity, it remains unknown whether a single CSC-targeting therapy is sufficient to eradicate cancers. A deep understanding of the molecular mechanism underlying cellular plasticity in cancer niche environments will be helpful for producing better strategies for CSC-targeting cancer therapy.

### Stem cells and interspecies chimaeras

Recent progress with various organoids has demonstrated the enormous self-organizing capacity for PSCs to form whole tissues. However, it remains particularly challenging to build real organs in vitro. Xenograft chimeras provide a possible solution for generating real organs. Typically, a chimaera is defined as an organism composed of cells that derive from more than one zygote. Based on whether cell derivatives from two zygotes are from different or the same species, chimaeras can be categorized as interspecies or intraspecies, respectively. In SC research, an interspecies chimaera is generated by transplanting SCs from the donor into an animal recipient at different stages of development [255]. Currently, research on interspecies chimaeras has gained increasing attention among researchers and the public due to its potential for generating functional human organs. In the 1970s, Gardner and colleagues generated mouse-rat chimaeras in which the rat inner cell masses were transplanted into mouse blastocysts and then transferred to the mouse uterus. In these chimaeras, extensive rat-derived cells could be observed in the fetuses; however, very few rat cells were detectable in the adult chimaeras [255]. The first PSC-derived interspecies chimaeras were generated by injecting ESCs of the wood mouse *Apodemus sylvaticus* into *Mus musculus* blastocysts. Notably, viable chimaeras contained a wide variety of donor cells in all major organs, including germ cells, of the host [256]. Furthermore, H. Nakauchi and colleagues recently achieved great progress in rodent chimeras by injecting mouse PSCs into Pdx-1-deficient rat blastocysts, and rat-sized pancreata consist of mouse PSC-derived cells. Subsequently, islets isolated from chimeric pancreata were transplanted into mice with streptozotocin-induced diabetes. The chimera-derived islets efficiently normalized host blood glucose levels for over 370 days without immunosuppression. These data provide rigorous evidence of the therapeutic potential of PSC-derived tissues and organs in chimeras [257].

Similar to chimera generation via interspecies blastocyst complementation with naive rodent PSCs, naive hPSCs have the potential to generate interspecies chimeras for studying human development and producing functional human tissue. To date, many studies have investigated the generation of hPSC-derived interspecies chimeras; however, the human–mouse chimera is rather inefficient because only a few human cells were detected in few chimeric embryos [258, 259]. Consistently, in human-ungulate chimeras, even naive hPSCs robustly implanted in both pig and cattle preimplantation blastocysts, and the contribution of hPSCs to postimplantation pig embryos was very limited [260]. To improve the inefficiency of chimerism achieved from hPSCs, several important factors need to be considered. First, the chimera host should be evolutionarily closely related to humans. Second, the pluripotent status of human SCs should match the developmental timing of the host. Third, both host animal SCs and hPSCs should be modified for better survival of hPSCs and efficient integration of hPSCs into targeted organs and to minimize the contribution of hPSCs to unwanted host organs, especially to the CNS and reproductive system [255] (Fig. 5e). In addition to blastocyst complementation, alternative SCs and host complementation strategies should be developed to regenerate various tissues and cells for clinical application. The integumentary chimera, for instance, has been established to generate intact skin tissue and its appendage organs [24, 261]. This chimera transplants interspecies or intraspecies SCs to the skin incision of adult or newborn animal and should be termed “tissue complementation chimera”, providing a feasible and alternative method to regenerate functional tissues and cells and even mini organs, such as hair follicles (Fig. 5f).

## Concluding remarks

Heterogeneity is the hallmark of SCs in normal and early neoplastic tissues, and the hierarchy that is established among heterogeneous SCs seems to be strictly regulated by the niche environments. However, cellular plasticity renders the SC hierarchy reversable and provides an alternative cellular mechanism through which tissues can regenerate when SCs are damaged. In addition, the robust plasticity of nontargeted cancer cells observed in CRC cells challenges the strategy of CSC-targeting cancer therapy. Epigenetic modifications play a crucial role in the underlying mechanism of cellular plasticity, and relevant epigenetic patterns have been well dissected in the context of cell reprogramming and differentiation. However, a more comprehensive picture of in vivo epigenetic modification in normal and disease cells requires further investigation. Organoids open new avenues for human cancer models and are promising for drug discovery. In addition to the potential of patient-derived organoids in basic biological research and regenerative medicine, these organoids can be used as a relevant model for personalized cancer treatments. While SCs are used to generate differentiated functional cells and 3D organoids, they are also used to generate interspecies chimaeras and have carved out new paths for fundamental biology studies as well as potential applications in regenerative medicine.
